# Temporomandibular Joint Sound Frequencies and Mouth-Opening Distances: Effect of Gender and Age

**DOI:** 10.3390/jcm14134399

**Published:** 2025-06-20

**Authors:** Serdar Gözler, Ali Seyedoskuyi, Ayşe Apak, Tan Fırat Eyüboğlu, Mutlu Özcan

**Affiliations:** 1 Department of Prosthodontics, Faculty of Dentistry, Istanbul Atlas University, Istanbul 41275, Türkiye; sgozler291@gmail.com; 2 Dentistry Faculty, Istanbul Aydin University, Istanbul 34295, Türkiye; seyedaliseyedoskouei@stu.aydin.edu.tr; 3 Department of Prosthodontics, Faculty of Dentistry, Prosthodontist, Kocaeli Sağlık ve Teknoloji University, Kocaeli 41001, Türkiye; ayse.apak@kocaelisaglik.edu.tr; 4 Department of Endodontics, Faculty of Dentistry, Medipol University, Istanbul 34083, Türkiye; 5 Clinic of Masticatory Disorders and Dental Biomaterials, Center for Dental Medicine, University of Zurich, 8006 Zurich, Switzerland; mutluozcan@hotmail.com

**Keywords:** age, clinical trial, gender, joint vibration analysis, temporomandibular disorders, temporomandibular joint, vibration frequencies

## Abstract

**Background/Objectives:** Temporomandibular joint disorders (TMDs) affect the temporomandibular joint and associated structures of the stomatognathic system. Joint Vibration Analysis (JVA) is a non-invasive method used to assess TMJ dysfunction through vibration frequencies. This study aimed to explore how age and gender influence TMJ vibration characteristics, hypothesizing that these factors may affect diagnostic accuracy in TMJ evaluations. **Methods**: This cross-sectional study includes 251 participants (143 females and 108 males) aged 10 to 30 years. TMJ evaluation used JVA to assess range of motion, integral values, and frequency distributions over and under 300 Hz. Participants with a history of TMJ disorders or significant maxillofacial trauma were excluded. Statistical analysis was conducted using employing Kolmogorov–Smirnov tests for data distribution, Kruskal–Wallis test for group comparisons, and Pearson correlation test for variable relationships. **Results**: Significant gender differences in range of motion (ROM) were observed, with males exhibiting higher values (*p* = 0.005). Age notably influenced vibration frequencies, particularly in total integral values (TIL and TIR) and frequency distributions around 300 Hz, suggesting links to degenerative changes. Females showed more pronounced age-related effects on vibration parameters. However, gender did not greatly affect vibration characteristics across all frequency bands, indicating that other factors also impact TMJ function. **Conclusions**: Age and gender significantly influence TMJ vibrations and the interpretation of JVA in clinical settings. Personalized approaches considering these demographic factors may enhance the accuracy of TMJ dysfunction diagnoses.

## 1. Introduction

Temporomandibular disorders (TMDs) encompass a range of symptoms affecting the temporomandibular joint (TMJ) and associated structures of the stomatognathic system. Stress is a significant factor that can increase tension within the masticatory system and alter mandibular biomechanics, potentially leading to joint sounds. It can also exacerbate TMDs through the mediation of bruxism. TMDs occur in about 34% of the population, and bruxism exists with TMDs in up to 18% of individuals [1]. The key manifestations of TMDs include pain in the chewing muscles and around the ear, intra-articular sounds during jaw movement, restricted movement, and deviation during mouth opening [2,3]. While pain often serves as the predominant symptom, it can arise from various structures within the masticatory system, and limitations in jaw movement are typically attributed to numerous underlying factors [4,5].

Intra-articular sounds, a notable feature of TMDs, specifically refer to sounds originating from within the joint, where no other areas of the stomatognathic system are associated with pathological sounds [6,7]. Evaluating these sounds is essential for accurate diagnosis, and several methods have been proposed for this purpose, including auscultation, sonography, vibratography, and Joint Vibration Analysis (JVA) [8,9]. The vibrations generated within the TMJ can provide critical insights into the nature and severity of the disorders present [10,11].

JVA is known for its reliability in providing quick and accurate diagnoses of TMJ anomalies [12,13]. During evaluation, a frequency of 300 Hz is used as a reference; frequencies below indicate soft tissue issues, while those above suggest hard tissue problems [14,15]. The Research Diagnostic Criteria for Temporomandibular Disorders (RDC/TMDs) categorize intra-articular discomfort into four frequency ranges: (1) 0–20, (2) 20–80, (3) 80–300, and (4) above 300 Hz [16]. The Diagnostic Criteria for Temporomandibular Disorders (DC/TMDs), a revised and validated version of the RDC/TMDs, is the gold standard for clinical practice and research, and does not classify frequency values in that manner [17] and therefore, making the RDC/TMDs more appropriate for studies involving JVA data interpretation. Identifying the boundary points between these ranges is crucial but can pose diagnostic challenges. Evaluating frequencies near these limits requires further clinical evidence, potentially necessitating adjunctive imaging like MRI and/or CBCT, which may detract from JVA’s primary advantage of chairside diagnosis.

The four fundamental frequency bands serve as key indicators for assessing the type and severity of TMJ disorders. Values significantly outside these thresholds provide clear diagnostic guidance. However, readings that are marginally below or above the thresholds can lead to uncertainty in diagnosis. Instead of immediately resorting to advanced imaging, complementary indicators such as range of motion (ROM), along with age and gender factors, can significantly support clinical diagnosis. Recent evidence suggests that the age and gender of asymptomatic individuals may influence vibration characteristics in the joint, enhancing diagnostic reliability for those with TMD [9,18,19,20]. Research has shown that TMJ vibration amplitudes increase with age, indicating progressive degenerative changes over time [5,20]. Additionally, gender-specific variations in joint laxity and hormonal influences have been associated with different vibration profiles in males and females [3,18]. Nonetheless, these findings are inconsistent and require further investigation.

This study evaluates whether specific TMJ vibration parameters obtained through Joint Vibration Analysis (JVA), particularly ROM and frequency distributions, correlate with age and gender. Understanding these relationships may lead to more accurate interpretations of vibration values near transition range limits [21,22,23,24,25]. The aim of this study is to determine if JVA parameters, such as ROM and vibration frequencies, are significantly influenced by age and gender in asymptomatic individuals, refining diagnostic accuracy for TMJ dysfunction. The null hypothesis (H_0_) posits that no significant relationship exists between gender, age, and the recorded vibration frequencies during TMJ assessments. Should this hypothesis be rejected, it would imply that these demographic factors may influence the diagnostic accuracy of JVA, thus enhancing the interpretation of TMJ vibration data in clinical practice [26,27,28].

## 2. Materials and Methods

### 2.1. Study Design

This investigation was conducted as a single-center, single-arm, and single-blind clinical trial (cross-sectional). All clinical procedures adhered to the standards established in the Declaration of Helsinki. Ethical approval was secured from the University Clinical Research Ethics Committee (approval date: 2 June 2021, reference number: B.30.2.AYD.0.00.00-050.06.04/492). Furthermore, informed consent was obtained from all participants before their inclusion in the study.

### 2.2. Recruitment, Inclusion, and Exclusion Criteria

The present study involved 251 subjects capable of achieving an inter-incisal opening of 30 to 50 mm without restrictions. In adults, a normal maximal incisal opening typically ranges from 35 to 55 mm, with 30 to 50 mm often considered functional, especially in studies of temporomandibular joint (TMJ) function and disorders. [19,25]. The sample size was sufficient to detect differences at a statistical power of 90% and a moderate effect size. The evaluation encompassed the range of motion (ROM) and vibrations of the temporomandibular joint (TMJ). Exclusion criteria were strictly applied: individuals utilizing psychotropic or narcotic medications, regardless of medical oversight; those who regularly consumed painkillers or anti-inflammatory agents; individuals undergoing long-term treatment with cortisone or its derivatives, as well as persons who had sustained traumatic injuries to the maxillofacial region, were excluded. Furthermore, participants who had suffered the loss of organs and/or tissue in the maxillofacial area due to either medical or traumatic causes were not considered for inclusion. The criteria for inclusion mandated that participants be free from symptoms indicative of temporomandibular disorder (TMD), possess fully and/or prosthetically restored dentition, and demonstrate adequate mouth opening to facilitate the rotation of the condylar process. Participants were deemed asymptomatic based on self-reported absence of TMJ-related pain, joint noise, or functional limitation, supplemented by a clinical examination performed by experienced clinicians. However, standardized diagnostic instruments such as the RDC/TMD or DC/TMD Axis I questionnaires were not utilized to confirm asymptomatic status. After collecting patients’ medical and dental histories, a thorough physical examination was conducted. The Masseter and Temporal muscles were palpated, and any sounds from the temporomandibular joint (TMJ) were recorded. Assessments, including the Mallampati score, Bruxism score, presence of apnea, and the Epworth Sleepiness Scale, were documented. A Cone Beam Computed Tomography (CBCT) scan was also performed for diagnostic purposes.

### 2.3. Temporomandibular Joint Evaluation

To measure the intra-articular frequency values of the participants, a Joint Vibration Analysis (JVA) accelerator device (Bio-JVA, Bioresearch Assoc. Inc., Brown Deer, WI, USA) was utilized. Vibration receiver silicones were positioned on the skin above the center of joint rotation, specifically in front of the right and left tragus regions of the individual, as previously described [11,12,20]. The average frequency values were recorded by monitoring the opening and closing of the mouths seven to eight times, with the data subsequently documented on the computer. The following parameters were measured:

1. Range of Motion (ROM): The incisal distance from centric occlusion to the maximum open position (in millimeters) was assessed using a calibrated Vernier caliper with an accuracy of 1 mm. Subjects were instructed to open their mouths to the fullest extent, and the caliper was employed to measure the interincisal distance, defined as the distance between the edges of the upper and lower anterior central incisors. The study population comprised 143 females and 108 males.
2. Total Integral Value for Right (TIR) and Left (TIL): This parameter represents the area under the mean Fast Fourier Transform (FFT) frequency distribution, quantifying the total pressure wave activity over time in kilohertz (KHz). It serves as a primary indicator of overall vibration intensity.
3. Integral > 300 Hz for Right (<300R) and Left (<300L): This refers to the segment of the Total Integral attributed to frequencies exceeding 300 Hz, which is predominantly influenced by roughened surfaces (KHz). This aspect of the frequency distribution is susceptible to degenerative changes occurring within the joint.
4. Integral < 300 Hz for Right (>300R) and Left (>300L): This component encompasses the portion of the Total Integral attributed to frequencies below 300 Hz, primarily associated with disk movements (KHz). This segment of the frequency distribution is significantly affected by disk displacements, reductions, hypermobility, or generalized joint laxity (GJL).
5. Integral >300/<300 Ratio: When the ratio of these integrals (>300 Hz/<300 Hz) exceeds 0.3, it indicates the presence of degenerative disease in the joints.

The differences in these four parameters will be evaluated based on age and gender, highlighting the significance of intra-articular sounds in diagnostic processes.

### 2.4. Statistical Analysis

The data were analyzed using the IBM Statistical Package for the Social Sciences (SPSS) version 22 software program (IBM Corp., Armonk, NY, USA). The data distribution is evaluated using the Kolmogorov–Smirnov test. Group comparisons were made using the Kruskal–Wallis test, and pairwise comparisons using the Mann–Whitney U test. Previously, age was accepted as a continuous variable, and the Pearson correlation test was utilized to evaluate any correlation between age and gender. However, age 22 was recognized as the breaking point for ROM and frequency values during the test procedure. Therefore, age was also evaluated as an ordinal value, creating two groups: those aged 22 and below and those aged 23 and above. The association between age and gender as categorical variables was determined using the chi-squared test. All statistical tests were conducted at a 95% confidence level (*p* < 0.05).

## 3. Results

**Figure 1:**
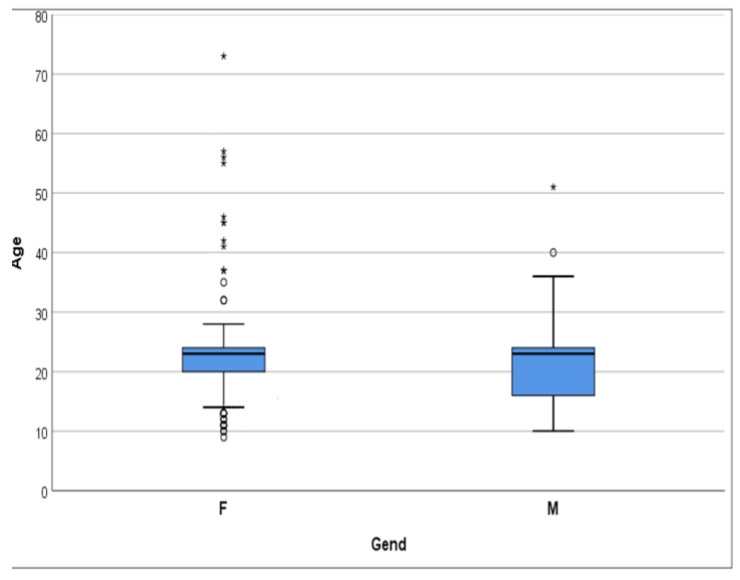
The distribution graph shows that the age group is predominantly under 30 years old (95%). * extreme outliers, ° mild outliers.

A total of 251 subjects participated in this study, including 143 females and 108 males. The age of participants ranged from 10 to 30 years, with an average age of 20.07 ± 4.85 years (Table 1, Figure 1). A statistically significant difference was observed in the age distribution between male and female participants, with the Kolmogorov–Smirnov test yielding a moderate difference between both genders (D = 0.17, *p* = 0.048), indicating that male participants (median: 23.00) tend to be younger than female participants (median: 20.00). Mann–Whitney U test also presented a difference between the age of both genders (*p* = 0.05). The correlation between age, as an ordinal or continuous variable, and gender was insignificant (*p* > 0.05, Table 2 and Table 3).

### 3.1. Range of Motion (ROM)

All ROM values according to age and gender variables are presented in Table 1. The analysis revealed a significant difference in ROM values between males and females, with males having significantly higher values (*p* = 0.005, small effect size). In both age groups, “22 and below” and “23 and above”, males presented significantly higher ROM values than females (*p* = 0.046, 0.038, respectively), with both comparisons showing small effect sizes (0.126 and 0.131). The differences in ROM values remained statistically significant within both age groups (*p* = 0.046 and *p* = 0.039, respectively, Table 4), again with small effect sizes, indicating a consistent but modest gender-related difference. Additionally, age as a continuous variable does not significantly correlate with ROM values for either gender (*p* > 0.05). This suggests that gender may influence ROM values independently of age, although the practical magnitude of this effect remains small.

### 3.2. Total Integral Left (TIL)

**Figure 2:**
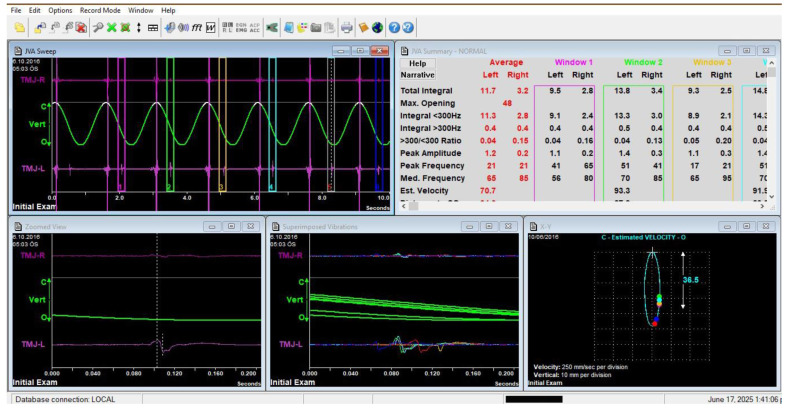
Frequencies of the internal vibrations were acquired by seven–eight maximum opening movements. Image of Joint Vibration Analysis (JVA) software (Biopak Ver. 8.7) showing patient data during measurement. It displays the vibration waveform and frequency spectrum during mandibular movement, illustrating the typical interface used for data collection and analysis in this study.

The Total Integral Left (TIL) variable demonstrated an asymmetric distribution. The mean value was measured at 8.16 ± 5.62, with a median of 6.2. Participants in both genders showed significantly higher TIL values in the “22 and below” group compared to the “23 and above” group (*p* = 0.01 for males, *p* = 0.000 for females), with small effect sizes in males (0.163) and very large effect size in females (inf). However, gender comparison within individual age groups presented no significant differences (*p* > 0.05, Table 4). The correlation between age as a continuous variable and TIL indicated a weak positive correlation (r = 0.218, *p* = 0.001), with a small effect size (0.22). The correlation was stronger in females (r = 0.287, small effect) than in males (r = 0.107, negligible), as shown in Table 2, Figure 2.

### 3.3. Total Integral Right (TIR)

Similar to the TIL, the Total Integral Right (TIR) variable also demonstrated an asymmetric distribution with a mean of 7.04 ± 5.80 and a median of 5.0. Both genders showed significantly higher TIR values in the “22 and below” group (*p* = 0.02 for males, *p* = 0.001 for females), with small effect sizes (0.147 and 0.208, respectively). However, comparing TIR values between males and females among individual age groups presented no significant differences (*p* > 0.05, Table 4). Age as a continuous variable was weakly correlated with TIR overall (r = 0.158, *p* = 0.012, small effect size), with slightly stronger correlation in females (r = 0.150) than males (r = 0.166), though both remained within the small effect range (Table 2).

### 3.4. <300 Hz Left (<300L) and <300 Hz Right (<300R)

Both the <300 Hz Left (c1) and <300 Hz Right (c2) variables demonstrated asymmetric distributions. The mean for c1 was 5.84 ± 4.39, and for c2, it was 5.22 ± 5.06 (Table 1). Significant differences were found between age groups in both genders for c1 (*p* = 0.01 in males, *p* = 0.000 in females) and c2 (*p* = 0.02 in males, *p* = 0.000 in females), with small effect sizes for males (0.163 for c1, 0.147 for c2) and very large effect sizes (inf) for females. However, gender comparison between individual age groups presented no significant difference in c1 or c2 values (*p* > 0.05, Table 4). As a continuous variable, age showed a weak positive correlation with c1 (r = 0.229, *p* = 0.000, small effect size) and c2 (r = 0.159, *p* = 0.012, small effect size). The correlation was stronger in females (r = 0.307 and 0.158) than in males (r = 0.106 and 0.159), consistent with small effect sizes (Table 2).

### 3.5. >300 Hz Left (>300L) and >300 Hz Right (>300R)

Both the >300 Hz Left (d1) and >300 Hz Right (d2) variables displayed asymmetric distributions. The mean value for d1 was 0.82 ± 0.67, while for d2, it was 0.84 ± 0.67. For d1, a significant difference between age groups was found only in females (*p* = 0.034, small effect size = 0.134), while d2 showed no significant differences in either gender (*p* > 0.05). Gender comparison between individual age groups showed no significant differences in d1 or d2 values (*p* > 0.05, Table 4). Age did not significantly correlate with either d1 (r = 0.055, *p* > 0.05) or d2 (r = 0.098, *p* > 0.05), both showing negligible effect sizes, with similar patterns across genders (Table 2).

### 3.6. Ratio Left and Ratio Right

The values of Ratio Left (e1) and Ratio Right (e2) were presented in Table 1. For males, there was no significant difference in e1 or e2 between the two age groups (*p* > 0.05), and effect sizes were negligible (0.053 and 0.048). For females, a significant difference was found in both e1 and e2 values between age groups (*p* = 0.000 for both), with very large effect sizes (inf).

Gender comparison between individual age groups presented no significant differences in e1 or e2 (*p* > 0.05, Table 4). Age showed no significant correlation with e1 (r = −0.081, *p* > 0.05) or e2 (r = −0.073, *p* > 0.05) in males. However, in females, there was a significant negative correlation with both e1 (r = −0.298, *p* = 0.000) and e2 (r = −0.285, *p* = 0.000), corresponding to small effect sizes. Overall, a small negative correlation was found across the entire sample for both e1 (r = −0.198, *p* = 0.001) and e2 (r = −0.188, *p* = 0.002) (Table 2).

## 4. Discussion

This study investigated the relationship between gender, age, and the vibration frequencies of the temporomandibular joint using Joint Vibration Analysis, an emerging diagnostic tool for assessing TMJ dysfunctions. Based on these results, the null hypothesis positing no significant relationship between gender, age, and the recorded vibration frequencies during TMJ assessments can be partially rejected. The findings indicate that gender significantly influences ROM values with small effect sizes, regardless of age, and age has a weak to small but statistically significant effect on TIL, TIR, <300 Hz, and >300 Hz values, especially in females, where effect sizes were mostly small to medium. Age-related changes in Ratio Left and Ratio Right values are significant in females with medium to large effect sizes but not in males. These results imply that demographic factors, particularly age and gender, have a modest but meaningful influence on the diagnostic accuracy of JVA. Therefore, incorporating these factors into the interpretation of TMJ vibration data can enhance clinical practice and improve the accuracy of TMJ dysfunction diagnoses.

The influence of age on TMJ vibrations aligns with previous research suggesting that TMJ function deteriorates over time due to physiological wear, reduced synovial lubrication, and degenerative changes in the articular cartilage [3,4]. Several studies have also reported similar findings, demonstrating a direct correlation between aging and increased intra-articular vibrations, which may indicate progressive joint degeneration [5,11]. Our results further reinforce these observations, as the increase in integral values in older individuals shows weak to small positive correlations (effect sizes ~0.16–0.23), suggesting a higher prevalence of roughened joint surfaces, possibly due to osteoarthritic changes.

Gender differences in TMJ function have been widely discussed in the literature. Our study supports prior findings indicating that females exhibit lower ROM values than males, potentially due to variations in muscle physiology, hormonal influences, and joint ligament elasticity [3,18,24]. Earlier studies have emphasized the role of estrogen in TMJ function, with evidence suggesting that estrogen receptors within the joint may affect ligament laxity and inflammatory responses [7,26]. This may help explain why females tend to experience higher rates of TMJ disorders and reduced ROM compared to males. Notably, the effect size for ROM gender differences was small (~0.13), indicating a modest but consistent gender effect. These observations align with those of Kondrat et al. (2018), who found that males had increased ROM and proposed that variations in joint morphology and the strength of masticatory muscles might play a role in this difference [19]. Additionally, Demir (2022) noted gender-related differences in MRI-based TMJ diagnostics, further underscoring the influence of hormonal and anatomical factors on joint mechanics [18]. However, unlike the findings by Matos et al. (2021) and Sharma et al. (2017), which identified more significant vibration abnormalities in symptomatic females, our asymptomatic group did not show meaningful gender-based frequency differences [7,12]. This highlights the importance of considering the specific context of the population when interpreting Jaw Vibrational Analysis (JVA) data.

Another significant finding of our study is the variation in vibration frequencies below and above 300 Hz. The Research Diagnostic Criteria for Temporomandibular Disorders (RDC/TMDs) classify frequencies below 300 Hz as indicative of soft tissue-related issues, such as disk displacement. In comparison, frequencies above 300 Hz are associated with degenerative changes, including osteoarthritic alterations. Our results demonstrate a significant increase in high-frequency vibrations (>300 Hz) among older individuals, consistent with findings in previous studies [7,11], which identified similar patterns in patients with TMJ dysfunction. However, the effect sizes for >300 Hz correlations with age were generally small (around 0.10), suggesting these changes, while significant, are subtle. This suggests that JVA could be a valuable, non-invasive tool for detecting early degenerative changes before they manifest as clinical symptoms.

Interestingly, our data also suggest that while age significantly influences TMJ vibrations, the impact of gender is more nuanced. While females exhibited lower ROM values, their overall vibration characteristics did not differ significantly from males except in specific frequency bands. Effect sizes for gender differences in vibration parameters were generally small or non-significant, indicating minimal clinical differences. This finding contrasts with a previous study, which reported greater TMJ sound intensity and altered vibration patterns in females compared to males [13]. This discrepancy may be attributed to differences in study populations, sample sizes, or variations in recording methods.

Our findings highlight the importance of considering the age-related biomechanical changes when evaluating TMJ function. However, the variability in vibration patterns suggests that factors beyond age and gender, such as occlusal patterns, parafunctional habits, and masticatory muscle strength, could contribute to TMJ dysfunction. Therefore, individual anatomical variations should also be accounted for. Previous studies have suggested that individuals with high masticatory activity, such as those who frequently chew gum or engage in bruxism, may exhibit altered vibration signatures regardless of age or gender [9]. Furthermore, the progression of TMJ dysfunction may not always follow a linear trajectory, as some individuals with mild degenerative changes may not exhibit significant symptoms. In contrast, others with minor structural alterations may cause severe discomfort. These observations underscore the importance of personalized diagnostic approaches that integrate JVA with patient-specific factors, including clinical history, occlusal analysis, and muscle activity assessment.

This study provides valuable information about the TMJ, but is not without limitations. The majority of the subjects were young, which limits generalizability to older populations where effect sizes and correlations may differ. The cross-sectional nature of the study does not allow for causal relationships between age, gender, and TMJ vibration parameters to be determined. Other potential confounding variables, such as hormonal fluctuations, bruxism, or parafunctional activity, were not measured or controlled, and these factors can also influence joint vibrations in asymptomatic individuals. Although participants were evaluated by experienced dental clinicians and only asymptomatic participants were included, standardized criteria such as RDC/TMD or DC/TMD criteria were not applied to validate TMD status. Furthermore, occlusal status was not measured and was not part of the inclusion criteria. These methodological limitations should be considered when interpreting the results. The variability in recording devices, such as accelerometers, could have influenced the findings. Standardization of measurement procedures is necessary for appropriate clinical diagnostics. Exclusion of symptomatic individuals also limits the generalizability of the results to TMD patients.

The findings suggest that JVA can be a valuable tool in TMJ diagnostics, particularly for the early detection of vibration changes associated with dysfunction. However, it should be combined with occlusal analysis, clinical history, and imaging methods like MRI or CBCT for a thorough assessment. Standardizing diagnostic thresholds is crucial for enhancing the clinical utility of JVA. Future research should investigate longitudinal studies to determine whether changes in vibration correlate with progressive TMJ pathology and how individual factors, such as parafunctional habits and occlusal discrepancies, influence these patterns. Developing personalized treatment strategies considering gender, age, and functional habits may improve clinical outcomes in TMJ management. In addition to its diagnostic value, the findings may have practical implications for early screening and targeted intervention in high-risk groups, potentially preventing chronic TMJ dysfunction. The observed age- and gender-related differences could hypothetically be linked to anatomical and physiological variations, such as joint laxity, hormonal influence, and cartilage resilience. This study supports the use of JVA in TMJ diagnostics, highlighting the need for early detection and individualized treatment approaches. Refining diagnostic criteria and incorporating JVA into a multimodal framework will strengthen its application in assessing TMJ function across diverse populations [29].

## 5. Conclusions

The results of this study indicated that age exerts a statistically significant but small to moderate effect on vibration frequencies, with older patients showing higher integral values suggestive of degenerative changes. Although males exhibited a greater range of motion than females, the effect size was small, indicating a modest gender effect. Vibration characteristics did not differ significantly between genders in most frequency bands, suggesting that while age and gender are important, their influence on TMJ vibration parameters is modest, and other factors likely contribute substantially. The study emphasizes the complex relationship between vibration signatures, joint biomechanics, and age, highlighting the need for a multifactorial approach to TMJ assessment using JVA.

## Figures and Tables

**Table 1:** Participant characteristics and TMJ parameters by gender. Mean ± SD values for age, range of motion (ROM), Total Integral Left (TIL), Total Integral Right (TIR), and frequency components (c1 < 300L, c2 < 300R, d1 > 300L, d2 > 300R, e1 RatioL, e2 RatioR) are shown for female, male and all participants.

	Female Mean ± SD			Male Mean ± SD			Total Mean ± SD		
	22 and Below	23 and Above	Total	22 and Below	23 and Above	Total	22 and Below	23 and Above	Total
Age	16.90 ± 3.75	24.09 ± 1.15	20.62 ± 4.52	15.70 ± 3.46	24.42 ± 1.75	19.33 ± 5.18	16.33 ± 3.65	24.22 ± 1.41	20.07 ± 4.85
ROM	42.67 ± 5.01	41.84 ± 7.78	42.34 ± 6.12	44.83 ± 5.00	45.11 ± 8.58	44.94 ± 6.69	43.70 ± 5.10	43.08 ± 8.21	43.40 ± 6.75
TIL	6.52 ± 5.05	10.48 ± 6.29	8.12 ± 5.87	6.80 ± 4.68	8.74 ± 5.24	7.61 ± 4.99	6.65 ± 4.86	9.82 ± 5.95	8.16 ± 5.62
TIR	6.04 ± 5.80	8.24 ± 6.20	7.12 ± 6.00	5.63 ± 3.89	8.58 ± 6.68	6.86 ± 5.41	5.84 ± 4.97	8.36 ± 6.36	7.04 ± 5.80
c1 < 300L	5.66 ± 4.41	9.48 ± 5.85	7.12 ± 5.13	6.03 ± 4.40	7.77 ± 4.64	6.76 ± 4.56	5.84 ± 4.39	8.83 ± 5.47	7.26 ± 5.14
c2 < 300R	5.14 ± 5.20	7.12 ± 5.42	6.02 ± 5.31	5.33 ± 4.88	7.45 ± 5.18	6.23 ± 5.03	5.22 ± 5.06	7.27 ± 5.32	6.11 ± 5.19
d1 > 300L	0.85 ± 0.79	1.01 ± 0.78	0.93 ± 0.79	0.78 ± 0.51	0.96 ± 1.04	0.86 ± 0.78	0.82 ± 0.67	0.99 ± 0.88	0.90 ± 0.78
d2 > 300R	0.89 ± 0.81	1.11 ± 1.20	0.98 ± 0.98	0.80 ± 0.48	1.10 ± 1.04	0.92 ± 0.78	0.84 ± 0.67	1.10 ± 1.14	0.97 ± 0.93
e1 RatioL	0.18 ± 0.09	0.12 ± 0.08	0.15 ± 0.09	0.15 ± 0.08	0.14 ± 0.10	0.15 ± 0.09	0.17 ± 0.09	0.13 ± 0.09	0.15 ± 0.09
e2 RatioR	0.23 ± 0.12	0.16 ± 0.09	0.20 ± 0.11	0.21 ± 0.11	0.18 ± 0.10	0.20 ± 0.11	0.22 ± 0.12	0.17 ± 0.09	0.20 ± 0.11

**Table 2:** Pearson correlation coefficients and significance levels for age as a continuous variable and range of motion (ROM) and other vibration frequencies (b1 Total_INTGRL_L (TIL), b2 Total_INTGRL_R (TIR), c1 < 300L, c2 < 300R, d1 > 300L, d2 > 300R, e1 RatioL, e2 RatioR).

	Age Correlation	ROM	b1 TIL	b2 TIR	c1 < 300L	c2 < 300R	d1 > 300L	d2 > 300R	e1 RatioL	e2 RatioR
TOTAL	Pearson Correlation	−0.054	0.218 **	0.158 *	0.229 **	0.159 *	0.057	0.098	−0.198 **	−0.188 **
	Sig. (2-tailed)	0.397	0.001	0.012	0.000	0.012	0.366	0.123	0.002	0.003
	Effect size	−0.0537	0.2231	0.1595	0.2353	0.1609	0.0574	0.0980	−0.2016	−0.1913
MALE	Pearson Correlation	0.001	0.107	0.166	0.106	0.159	0.056	0.156	−0.081	−0.073
	Sig. (2-tailed)	0.989	0.270	0.086	0.274	0.101	0.568	0.107	0.404	0.450
	Effect size	0.001	0.108	0.168	0.107	0.161	0.056	0.158	−0.081	−0.074
FEMALE	Pearson Correlation	−0.054	0.287 **	0.150	0.307 **	0.158	0.048	0.055	−0.298 **	−0.285 **
	Sig. (2-tailed)	0.520	0.001	0.074	0.000	0.059	0.570	0.512	0.000	0.001
	Effect size	−0.054	0.299	0.151	0.323	0.160	0.048	0.055	−0.312	−0.297

** *p* < 0.05 in total, and one of the genders. * *p* < 0.05 only in total.

**Table 3:** Differences (Mean ± SD) and *p*-values between different age groups in each gender.

	Gender	Age Group Difference 22 and Below vs. 23 and Above (Mean ± SD) (KHz)	*p*	Effect Size
ROM	Males	+2.5 ± 0.5	0.046 *	0.126
	Females	+2.3 ± 0.4	0.038 *	0.131
TIL	Males	−1.8 ± 0.3	0.01 *	0.163
	Females	−2.1 ± 0.4	0.000 *	inf
TIR	Males	−1.5 ± 0.3	0.02 *	0.147
	Females	−1.9 ± 0.4	0.001 *	0.208
<300L	Males	−1.2 ± 0.2	0.01 *	0.163
	Females	−1.4 ± 0.3	0.000 *	inf
<300R	Males	−1.1 ± 0.2	0.02 *	0.147
	Females	−1.3 ± 0.3	0.000 *	inf
>300L	Males	−0.2 ± 0.1	0.067	0.116
	Females	−0.3 ± 0.1	0.034 *	0.134
>300R	Males	−0.1 ± 0.1	0.089	0.107
	Females	−0.2 ± 0.1	0.072	0.114
e1	Males	−0.05 ± 0.01	0.404	0.053
	Females	−0.08 ± 0.02	0.000 *	inf
e2	Males	−0.04 ± 0.01	0.450	0.048
	Females	−0.07 ± 0.02	0.000 *	inf

* *p* < 0.05.

**Table 4:** Differences and *p*-values between genders in each age group separately for each variable.

	Age Group	Gender Comparison Males vs. Females (Mean ± SD) (KHz)	*p*	Effect Size
ROM	22 and below	+1.81 ± 7.14	0.046 *	0.126
	23 and above	+4.71 ± 11.96	0.039 *	0.130
TIL	22 and below	+0.59 ± 6.59	0.190	0.083
	23 and above	−1.42 ± 5.99	0.310	0.064
TIR	22 and below	+0.4 ± 0.1	0.174	0.086
	23 and above	+0.3 ± 0.1	0.576	0.035
<300L	22 and below	+0.3 ± 0.1	0.658	0.028
	23 and above	+0.2 ± 0.1	0.918	0.006
<300R	22 and below	+0.2 ± 0.1	0.658	0.028
	23 and above	+0.1 ± 0.1	0.918	0.006
>300L	22 and below	+0.1 ± 0.1	0.658	0.028
	23 and above	+0.1 ± 0.1	0.918	0.006
>300R	22 and below	+0.1 ± 0.1	0.658	0.028
	23 and above	+0.1 ± 0.1	0.918	0.006
e1	22 and below	+0.02 ± 0.01	0.658	0.028
	23 and above	+0.03 ± 0.01	0.918	0.006
e2	22 and below	+0.02 ± 0.01	0.658	0.028
	23 and above	+0.03 ± 0.01	0.918	0.006

* *p* < 0.05.

## Data Availability

Datasets used and/or analyzed during this study are available from the appropriate authors upon reasonable request.
